# Cattle Abortions and Congenital Malformations Due to Bluetongue Virus Serotype 3 in Southern Belgium, 2024

**DOI:** 10.3390/v17101356

**Published:** 2025-10-10

**Authors:** Laurent Delooz, Nick De Regge, Ilse De Leeuw, Frédéric Smeets, Thierry Petitjean, Fabien Grégoire, Claude Saegerman

**Affiliations:** 1 Regional Association for Animal Registration and Health (ARSIA) asbl, 5590 Ciney, Belgium; frederic.smeets@arsia.be (F.S.); thierry.petitjean@arsia.be (T.P.); fabien.gregoire@arsia.be (F.G.); 2 Research Unit of Epidemiology and Risk Analysis Applied to Veterinary Science (UREAR-ULiège), Fundamental and Applied Research for Animals & Health (FARAH) Center, Faculty of Veterinary Medicine, University of Liege, 4000 Liege, Belgium; 3 Service of Exotic and Vector-Borne Diseases, National Reference Laboratory for Bluetongue, Sciensano, 1080 Brussels, Belgium; nick.deregge@sciensano.be (N.D.R.); ilse.deleeuw@sciensano.be (I.D.L.)

**Keywords:** bluetongue virus serotype 3, BTV-3, abortion, surveillance, transplacental transmission, congenital malformation, cattle, epidemiology, Belgium

## Abstract

In July 2024, bluetongue virus serotype 3 (BTV-3) was first detected in southern Belgium, marking the onset of a major epidemic wave. This study documents, for the first time in Belgium, the ability of BTV-3 to cross the placental barrier in cattle, causing abortions and congenital central nervous system malformations. Abortion cases from January to December 2024 were monitored through the national abortion protocol, which mandates reporting and laboratory investigation (i.e., the year of emergence and the three previous years as the baseline data set). Among 5,751 reported abortions, 903 foetuses were tested by PCR, revealing widespread BTV-3 circulation. The first malformed PCR-positive foetus was recorded in mid-August, four weeks after a sharp increase in abortion rates. Lesions such as hydranencephaly were confirmed in PCR-positive foetuses, with a malformation rate of 32.24% in affected herds from weeks 36 to 52 (i.e., 22 times higher than in previous years). Gestational stage analysis indicated that congenital lesions were most frequent following infection between 70 and 130 days of gestation. Based on the observed gross lesions and the timing of abortion, it was deduced that the earliest maternal infections likely occurred in February–March 2024, implying low-level winter BTV-3 circulation before the official detection of the epidemic wave. These findings highlight the epidemiological value of systematic abortion monitoring as an early warning system tool and highlight the inadequacy of relying solely on clinical surveillance in adult ruminants. The abrupt emergence of BTV-3 across the territory without a gradual spatial spread underscores the need for anticipatory control strategies. Strategic, multivalent vaccination campaigns and enhanced abortion surveillance are critical to mitigate similar reproductive and economic losses in future bluetongue outbreaks.

## 1. Introduction

Bluetongue (BT) is an infectious disease caused by an orbivirus belonging to the Orbivirus genus within the family Sedoreoviridae, with a segmented genome consisting of 10 dsRNA segments encoding for seven structural proteins and at least four non-structural proteins [1]. A total of 24 serotypes of bluetongue virus (BTV) have been recognised and are notifiable to the World Organisation of Animal Health. The virus is transmitted by *Culicoides* biting midges, which restricts its distribution to areas where competent vectors occur. Since 1998, BTV serotypes 1, 2, 3, 4, 6, 8, 9, 11, and 16 have been reported in Europe. In 2006, BTV serotype 8 (BTV-8) emerged unexpectedly in northern Europe throughout a region including Belgium, France, Germany, Luxembourg, and the Netherlands [2,3].

In September 2023, bluetongue virus serotype 3 (BTV-3) emerged in the Netherlands, causing severe clinical signs in ruminants [4]. A few weeks later in October, the virus was detected in six different herds in the province of Antwerp, in the northern part of Belgium, which borders the Netherlands. BTV-3 also emerged in Germany and was detected in ruminants and in pools of *Culicoides* [5]. The BTV-3 also emerged subsequently in England in November 2023 [6]. Virus replication within *Culicoides* biting midges is known to be temperature dependent [7]. The unfavourable climatic conditions during the winter of 2023–2024 in Belgium significantly limited both viral replication and transmission. These same conditions were also unsuitable for vector activity, which is closely linked to environmental factors such as temperature and humidity [8,9]. As a result, no new outbreaks of BTV were reported in either Belgium or Germany during that winter period [10]. However, ongoing climate change is expected to facilitate the overwintering of the virus, to prolong the period of vector activity, and to shorten the extrinsic incubation period, thereby increasing the risk of future outbreaks [11,12].

On 14 June 2024, a first reporting was confirmed in the Netherlands and two weeks later, in the south-eastern part of Belgium on 9 July 2024, i.e., the first outbreak of the year was detected, marking the beginning of a bluetongue epidemic wave [1]. BTV-3 was subsequently confirmed by PCR in multiple species in the southern part of Belgium, including cattle, sheep, goats, and camelids. This unprecedented spread raised concerns about the potential long-term impact of BTV-3 on livestock health and production.

The emergence and spread of BTV-3 in Europe underscore the critical importance of robust surveillance systems. The World Organisation for Animal Health (WOAH) has highlighted how climate change influences vector populations, thereby shifting disease patterns and facilitating the northward spread of bluetongue in Europe. This study describes the evolution of the infection, the consequences and the impact of the disease focusing on bovine abortions. In Europe, the reporting of the BTV is mandatory, the disease is classified C, D, and E in the Animal Health Law (AHL) and all suspect cases must be reported and analysed [13]. In addition, a surveillance programme called “Belgian abortion protocol” based on the mandatory reporting of abortions [14] allowed a monitoring of the disease. This protocol uses laboratory investigations and serves multiple objectives, including official disease monitoring and the detection of emerging pathogens. Within this framework and in response to the substantial economic losses, bovine abortions, and mortality documented during the previous emergence of BTV-8 in Belgium, BTV diagnostics were integrated into this protocol to enable the early detection and assessment of reproductive effects from subsequent serotypes, including BTV-3.

Some serotypes of BTV are able to cause congenital malformations like other viruses [15]. We hypothesised that BTV-3 can cross the bovine placenta, leading to abortion and congenital central nervous system malformations. Using the Belgian mandatory abortion–reporting system, we (i) documented temporal changes in abortion incidence in 2024; (ii) quantified PCR–confirmed BTV-3 detections in aborted foetuses with and without CNS malformations; and (iii) inferred the timing of maternal infection by aligning gestational stage, lesion type, and seasonal vector activity. These different pieces of information were combined and were useful for the understanding and the knowledge of BTV infection in European conditions to be better prepared for eventual future BTV infection.

## 2. Materials and Methods

### 2.1. Study Design and Passive Monitoring System

The regional association for animal registration and health from the southern part of Belgium, in Wallonia (ARSIA), monitors the presence of the disease and induced pathology in the necropsy room by experienced veterinary pathologists. Since 2011, the abortion protocol has included BTV analyses on aborted foetuses if suspicious congenital lesions are observed. The BTV congenital suspect observations are arthrogryposis and lesions on the central nervous system (CNS) as hydrocephaly, hydranencephaly, microcephaly, porencephaly, and cerebellar hypoplasia [15,16,17,18]. These gross lesions are registered in the Laboratory Information Management System (LIMS). While the pre-existing protocol targeted BTV PCR primarily to foetuses with congenital lesions or when the reporting veterinarian suspected BT, during the 2024 epidemic we also performed PCR on numerous foetuses without gross CNS lesions.

In the context of the Belgian passive surveillance programme for bovine brucellosis, a total of 18695 bovine abortion cases were collected from January 2021 to December 2024 and were included in this study (i.e., the year of emergence and the three previous years as the baseline data set). They originated from 4316 cattle farms distributed among all five Walloon provinces (Table 1). Since 3 October 2024, for budgetary reasons, the Belgian health authorities are required to adjust the abortion protocol until the end of the year; only testing for brucellosis and BVD was still mandatory. During this period, breeders still had the option to report suspect cases and test aborted foetuses for BTV-3, but at their own expenses.

**Figure 1:**
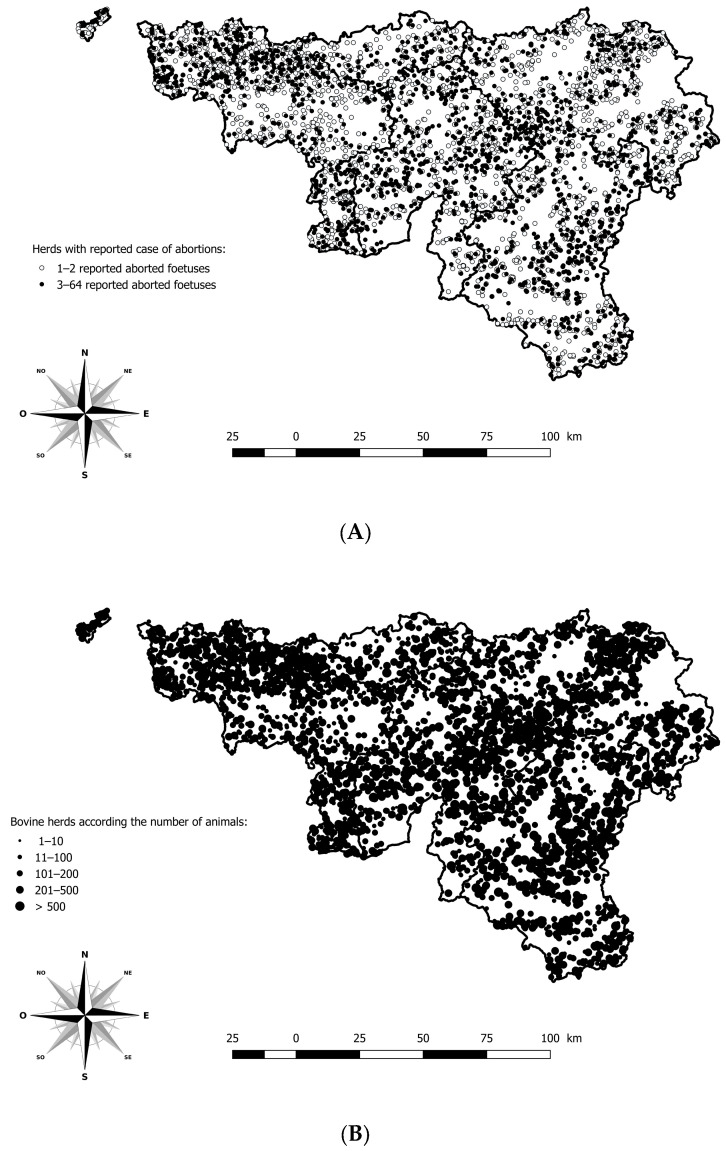
Geographical distribution of herds with a minimum of one aborted foetus reported and analysed in the southern region of Belgium, between 2021 and 2024. (**A**) Geographical distribution of herds according to the number of aborted foetuses reported. (**B**) Geographical distribution of bovine herds according to the number of animals. Legend: The five provinces of the southern part of Belgium are delimited by a solid line (i.e., Hainaut, Brabant Wallon, Liège, Luxembourg, and Namur).

Information issued from the anamnesis, such as sampling date, herd identification number, gestational stage, and cattle breed was registered in the LIMS or in an Access^®^ database. Data from the monitoring and surveillance system were available to describe clinical signs and pathological findings related to BTV-3 in cattle. The geographical location of each case of abortion was possible using the Lambert coordinates and the Belgian cattle identification and movement traceability system (SANITRACE) (Figure 1).

### 2.2. Laboratory Analyses

#### 2.2.1. Analyses on Necropsied Animals Including Foetuses

A standardised panel of analyses was first applied to perform the laboratory diagnosis of bovine abortion on submitted foetuses. A direct and/or indirect detection of pathogens was performed, including bacteria (*Anaplasma phagocytophilum*, *Brucella* spp., *Campylobacter* spp., *Coxiella burnetii*, *Leptospira borgpetersenii* and interrogans serovar Hardjo, *Listeria monocytogenes*, *Neospora caninum*, *Salmonella* spp., and *Ureaplasma diversum*), viruses such as bluetongue virus serotype 3 and 8 (BTV-3, BTV-8), bovine herpesvirus 4 (BoHV-4), bovine viral diarrhoea virus (BVDV), and Schmallenberg virus, several mycotic agents, and many other opportunistic bacteria (Table 2).

A specific panel of analyses was applied by the pathologists during the necropsy to perform the laboratory diagnosis of BT and to monitor all suspicious cases. The congenital suspicious lesions are arthrogryposis and lesions on the CNS as hydrocephaly, hydranencephaly, microcephaly, porencephaly, and cerebellar hypoplasia as described in numerous studies [15,18,19].

#### 2.2.2. Analyses on Living Animals

BTV Antibody ELISA on serum samples: The diagnosis of BTV in the serum was performed using an ELISA for the detection of antibodies against BTV (PanBTV, no specific serotype) in bovine serum according to the manufacturer’s instructions (kit ID Screen^®^ Bluetongue Competition, from Innovative Diagnostics, Grabels, France).

BTV PCR on blood samples (including BTV-3 and BTV-8 detection): The diagnosis of BTV in whole blood (EDTA) was performed using a commercial real-time PCR for the detection of BTV panserotypes (PanBTV, Kit ADIAVET^®^ BTV REALTIME, BioX Diagnostics, Rochefort, Belgium) according to the manufacturer’s instructions.

BTV PCR on aborted foetuses (including BTV-3 and BTV-8 detection): During the necropsy, spleen, kidney, liver, and placenta fragments were sampled on each abortion case and stored at −20 °C. BTV PCR analysis was performed on the spleen sample from the foetuses with suspicious congenital lesions or at the request of the veterinarian who declared the abortion following clinical suspicion of the dam. The real-time PCR tests were performed with different methods according to the targeted serotypes: BTV pan-serotypes (kit ADIAVET^®^ BTV REALTIME, from BioX Diagnostics, Rochefort, Belgium), BTV-3 (kit ID Gene™ Bluetongue genotype 3 Duplex, from Innovative Diagnostics, Grabels, France), and homemade BTV-8 PCR in Sciensano [20]. According to the manufacturer’s instructions, a sample was considered positive with a threshold cycle (=Ct) value lower than 45 for the pan-serotypes PCR and lower than 40 for the other PRC methods. The ADIAVET^®^ kit, tested against all reference BTV serotypes (1–24) and evaluated with the reference technique provided by AFSSA–LERPAZ, showed 100% analytical sensitivity and no cross-reactions with EHDV or other viruses/bacteria. For the BTV-3 kit, in silico analysis using sequences from the NCBI nucleotide database confirmed 100% specificity for a highly conserved segment of the BTV-3 genome, with no cross-reactivity detected in analytical testing. Indeed, both methods provide high analytical specificity, with the BTV-3 assay ensuring reliable genotype identification without interference from other BTV serotypes or other related orbiviruses.

In this study, virus detection in foetal tissues was based on pan-BTV and BTV-3/BTV-8-specific PCR assays. No sequencing or genotyping of foetal isolates was performed, which we acknowledge as a limitation of this work.

### 2.3. Statistical Analyses

To test the effect of the congenital lesions of the CNS and the month of detection on the ratio between PCR positive and negative, a linear regression was used. A *p*-value less than 0.05 was considered as significant.

The starting point of the epidemic wave was identified using a test for structural break in time-series data [21]. Firstly, the weekly average number of reported bovine aborted foetuses was estimated using data from the 3 years before the epidemic (as a baseline). Secondly, the weekly difference between the baseline data and the data for the year 2024 was calculated. Thirdly, this difference was computed using a regression and the Stata function estat sbsingle test for a structural break after estimation with regression (Stata SE 14.2, College Station, TX, USA). This technique is known to be robust in case of unknown forms of heteroskedasticity. A *p*-value less than 0.05 in the Wald test is indicative of the existence of a specific structural break. The same analysis was used to identify the week related to an important change in the rate of suspicious congenital lesions on aborted foetuses in 2024 and in the rate of congenital malformations.

The rate of congenital lesions in aborted foetuses in function of the month of pregnancy of cattle was computed using the locally weighted scatterplot smoothing method (Lowess method) that is a robust locally weighted regression [22].

The number of abortions reported in function of the breed, the period of the year (before and after the first reported in August), and the presence of congenital malformations was analysed using the negative binomial regression.

## 3. Results

### 3.1. Clinical Presentation

**Figure 2:**
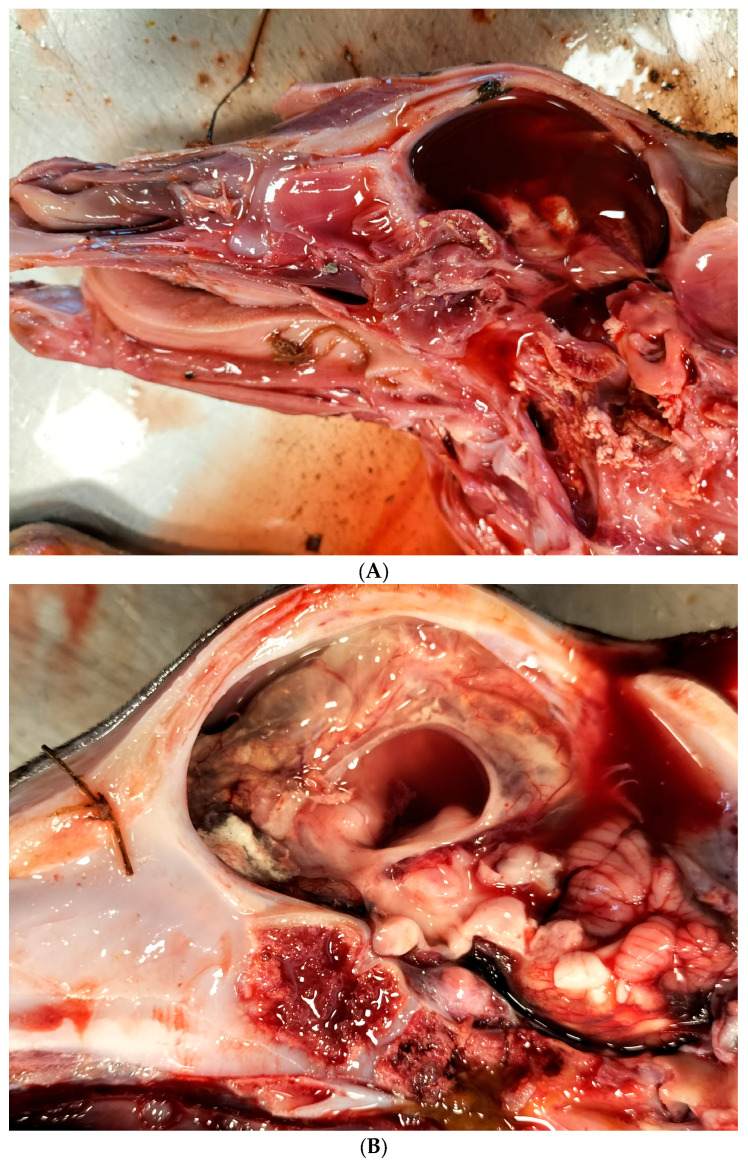

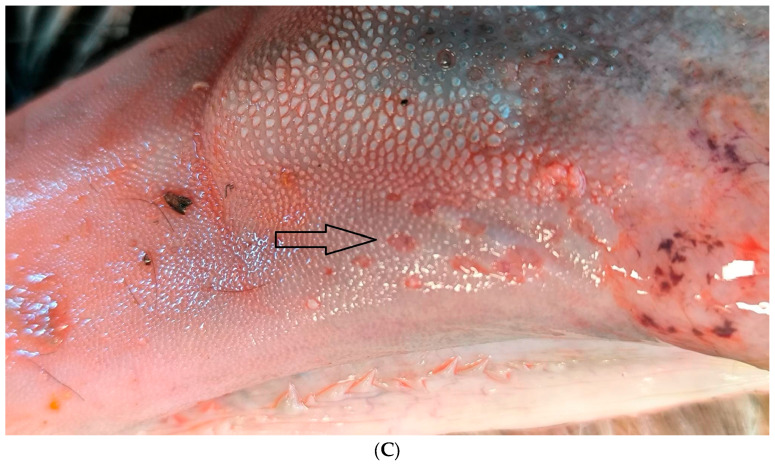
Typical congenital lesions observed in the necropsy room in BTV-3 confirmed foetuses. (**A**) Hydranencephaly on 7-month-old bovine aborted foetus. (**B**) Hydranencephaly on 8-month-old bovine aborted foetus. (**C**) Ulcerations on the tongue of a 9-month-old bovine aborted foetus.

According to the abortion monitoring, the main clinical signs reported during the epidemic was first, at the beginning of the month of August 2024 (week 31), an increase in the body temperature of the dam accompanied by abortions without congenital lesions (Appendix A). Two weeks later, CNS congenital lesions on aborted foetuses including hydranencephaly (Figure 2A,B), hydrocephaly, microcephaly, porencephaly, and mucosal ulcerations (Figure 2C) at the level of the tongue were observed on an aborted foetus.

### 3.2. Monitoring of BTV Using the Abortion Protocol

**Figure 3:**
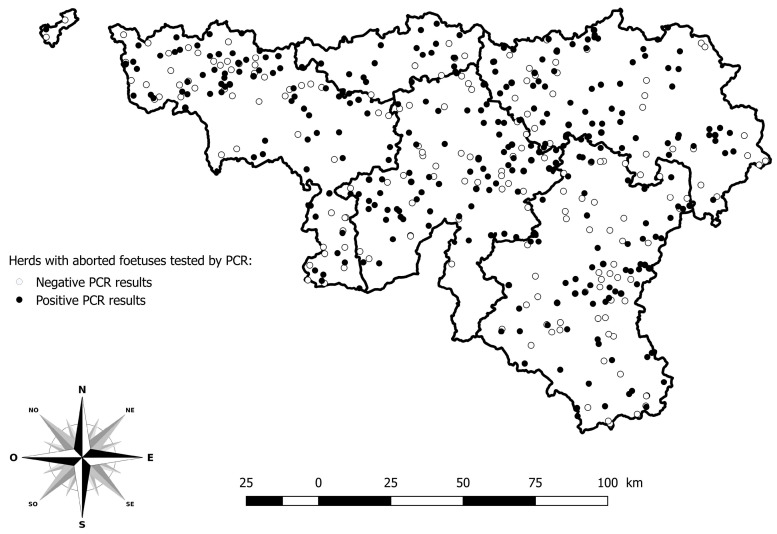
Geographical distribution of herds with aborted foetuses tested by PCR for BTV. Legend: The five provinces of the southern part of Belgium are delimited by a solid line (i.e., Hainaut, Brabant Wallon, Liège, Luxembourg, and Namur).

Among the 5751 abortion cases reported in 2024, 903 were tested by PCR BTV among 484 herds. The herds with PCR positive results are distributed across the whole study area (Figure 3).

The monthly distribution of the BTV PCR results according to the congenital lesions of the CNS is presented in Table 3.

A linear regression using the ratio between the number of positive and negative PCR as the dependent variable and both the congenital lesions of the CNS and the month as independent variables indicates a significantly higher ratio in the case of presence of congenital lesions (*p*-value = 0.043) both in August and September 2024 (*p*-value = 0.003).

At the end of week 33, on 17 August 2024, the first aborted foetus presenting CNS malformations and tested PCR-positive was recorded in the province of Hainaut. The case involved a 7-month-old Holstein foetus exhibiting hydranencephaly.

**Figure 4:**
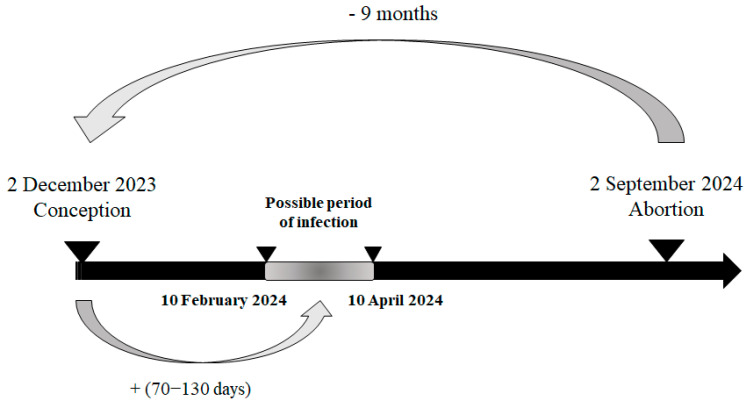
Most plausible scenario of the kinetic of infection for the first infected malformed aborted foetus in 2024.

Based on the reported gestational stage of the necropsied foetuses, it was possible to estimate their date of conception and, consequently, the likely at-risk period for infection. During week 36, on 2 September 2024, the foetus with the earliest estimated at-risk period was reported. This case concerned a 9-month-old Holstein foetus from Luxembourg province, also affected by hydranencephaly. The estimated date of conception was 2 December 2023, with the likely at-risk period for infection—between the 70th and 130th day of gestation—falling between 10 February 2024 and 10 April 2024, corresponding to epidemiological weeks 6 to 15 of 2024 (Figure 4).

**Figure 5:**
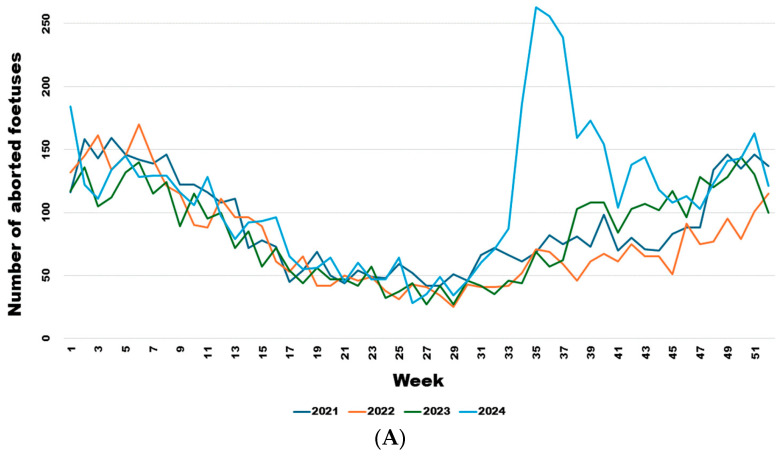

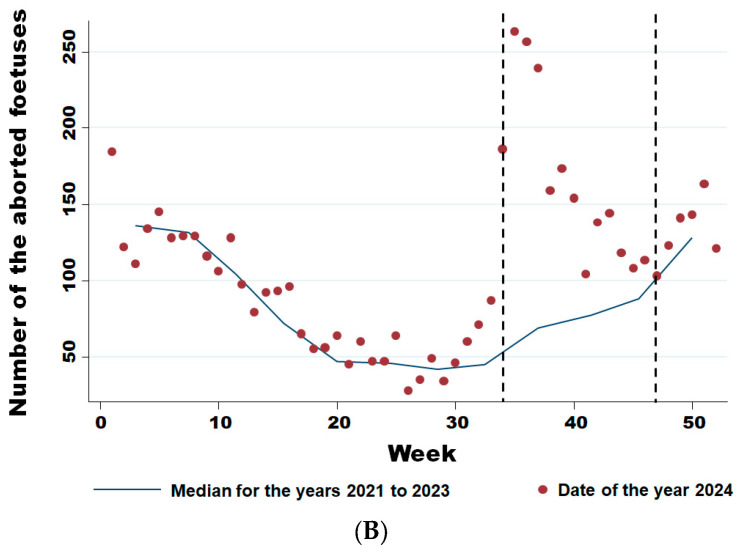
Weekly evolution of reported bovine aborted foetuses for the years 2021–2024 (**A**) and of the difference between the average bovine aborted foetuses during the 3 years before the epidemic and the number for the year 2024 (**B**). Legend: The first vertical dotted line corresponds to the starting break of the epidemic wave in week 34 (Wald test for a structural break = 126.17 and *p*-value < 0.0001). The second vertical dotted line corresponds to the end of the epidemic wave in week 47 (Wald test for a structural break = 8.26 and *p*-value = 0.016).

From week 34 onwards, the number of abortions increased significantly (Figure 5A), even though the virus had already been detected in southern Belgium for several weeks in adult animals but not in aborted foetuses. Comparing the weekly trend in the number of abortions in 2024 with the previous three years (Figure 5B), it is clear that this was a significant sharp increase in abortions that began in August (week 34) (Wald test for a structural break = 126.17 and *p*-value < 0.0001) and continued until the end of November (week 47) (Wald test for a structural break = 8.26 and *p*-value = 0.016).

**Figure 6:**
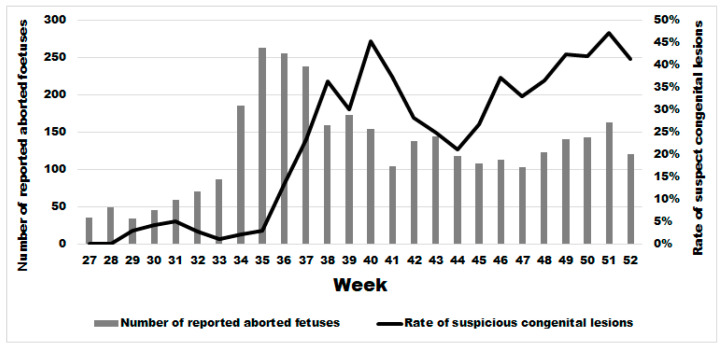
Weekly evolution of the number of reported bovine aborted foetuses and the rate of suspect congenital lesions on aborted foetuses in the second half of 2024.

Two weeks after the significant sharp increase in abortions at week 34, we observed a significant increase in the rate of malformations (Figure 6) starting at week 36 in comparison to the previous period (Wald test for a structural break = 15.41 and *p*-value = 0.0005). In the same year, from week 36 until week 52, the average rate of congenital malformations was 32.24% (95% IC: 30.41–34.11), being twenty-two times more than during the same period in the previous 3 years (OR 21.67 (95% CI: 17.06–25.54 with a *p*-value < 0.0001). No significant difference was observed in the rate of suspect congenital lesions depending on the cattle breed, i.e., meat, dairy or mixed (linear regression; *p*-value > 0.22). However, the starting break of this rate is statistically different and was at week 36 (Wald test for a structural break = 135.02 and *p*-value = 0.0001), 37 (Wald test for a structural break = 93.36 and *p*-value = 0.0001) and 39 (Wald test for a structural break = 11.87 and *p*-value = 0.044) for dairy, meat, and mixed cattle, respectively (Appendix B).

**Figure 7:**
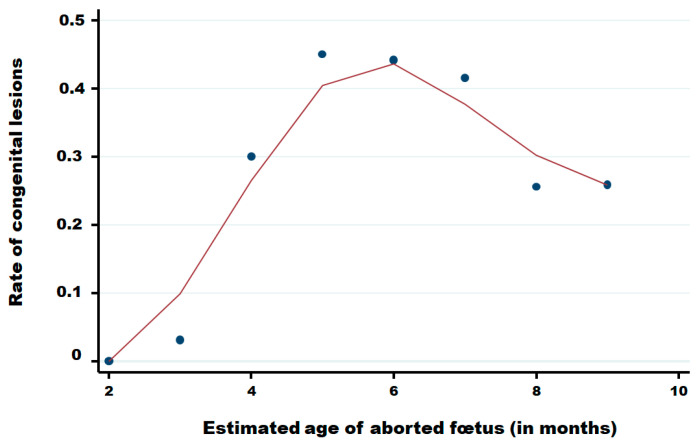
Rate of congenital lesions in aborted foetuses in function of the month of pregnancy of cattle. Legend: Points are observed data; the red solid line was obtained using the Lowess smoothing method [22].

Table 4 summarises the distribution of reported abortions by estimated gestational month, stratified by the presence of CNS congenital lesions and BTV PCR status. Among foetuses with CNS lesions *(n* = 806), PCR positivity was detected from month 3 through month 9, with the highest proportional positivity in mid-gestation: month 5, 29/82 (35.4%); month 6, 40/169 (23.7%); and month 7, 36/150 (24.0%); proportions declined thereafter in month 8, 21/120 (17.5%), and month 9, 42/255 (16.5%). Among foetuses without lesions (*n* = 1,694), PCR-positive detections were observed across the same gestational range but at lower proportions, i.e., month 5, 9/100 (9.0%); month 6, 6/213 (2.8%); month 7, 8/210 (3.8%); month 8, 29/347 (8.4%); and month 9, 61/727 (8.4%). Early gestation contributed a few cases (months 2–3; very small denominators), and “unknown” gestational age was rare (*n* = 13). Overall, PCR positivity was more frequent among foetuses with lesions (174/806, 21.6%) than among those without lesions (118/1,694, 7.0%) with an odds ratio of 3.68 (95% CI: 2.86–4.74), while the absolute number of cases increased with advancing gestation in both strata. Whereas few abortions are reported in the two first months of pregnancy, congenital lesions are observed from the third month until the ninth month of pregnancy with a clear increase in the fourth month, a higher level between the fifth and the seventh month of pregnancy and after, a decrease until the ninth month (Figure 7).

The number of abortions reported does not depend on the period before versus after the 1 August 2024 (negative binomial regression; *p*-value = 0.60). The number of abortions reported is significantly less important for mixed cattle herds (negative binomial regression; *p*-value = 0.01) and in case of congenital lesions (negative binomial regression; *p*-value = < 0.001) (Table 5).

## 4. Discussion

This study provides unique data on the monitoring of abortion cases during the BTV-3 epidemic in Wallonia, thereby contributing to a better understanding of the epidemiology of BTV-3. This study also provides the first conclusive field and epidemiological evidence that BTV-3 can cross the placental barrier, infect the foetus and cause congenital malformations in cattle. While previous studies or outbreaks of other serotypes, notably BTV-8, have demonstrated similar pathologies [15,16,18], this is the first report linking BTV-3 with such reproductive consequences. These findings are consistent with teratogenic profiles reported for orbiviruses (e.g., BTV–8) and for orthobunyaviruses (e.g., Schmallenberg, Akabane) in ruminants; BTV-3 itself is an orbivirus [23].

The results demonstrate a temporal association between the increase in the number of reported abortions and the detection of BTV-3 in aborted foetuses. From week 34 of 2024, abortion rates rose significantly, preceding the occurrence of CNS malformations in foetuses by two weeks. The 32.24% malformation rate among foetuses from BTV-3-positive herds during weeks 36–52 exceeds the background rates from previous years twenty-two-fold. Understanding the relationship between the gestational stage at which infection occurs and the subsequent development of congenital lesions such as hydranencephaly is essential for reconstructing the temporal dynamics of virus circulation. By aligning the estimated conception dates, gestational stages, and observed lesions with the known seasonal activity of *Culicoides* vectors [8], it becomes possible to infer when maternal infections most likely took place, even before the first officially reported cases.

The virus transmission is normally limited to those times of the year when adult insects are active [8]. The spatial spread of PCR-positive abortions across southern Belgium suggests widespread vector activity, likely sustained by favourable climatic conditions, a dense population of *Culicoides*, and a long-distance wind dispersal of *Culicoides* spp. estimated week by week [24].

The persistence of BTV across winters likely reflects multiple mechanisms [12]. Overwintering in long-lived parous female *Culicoides* has been documented in California (BTV-positive mid-winter midges) [25], although other mechanisms cannot be ruled out. In western Germany in 2023, BTV-3 was detected in *Culicoides* pools during the outbreak [5]. In Belgium, *Culicoides* activity/overwintering has been reported [26]. Vertical (transplacental) infection in cattle—documented here—may also contribute to early-season detection.

Regarding the transplacental transmission of the virus and its impact on the foetus depending on the stage of gestation, this study also allows us to suggest the most likely period of the first infections of the dams in Wallonia (between February and April 2024).

Thanks to the collected data, including the type of lesions observed, gestational stage, and the date of abortion, it was estimated that the first infection of a malformed PCR positive foetus occurred between epidemiological weeks 6 and 15 in the province of Luxembourg. Congenital malformations such as hydranencephaly occur when transplacental infection takes place between approximately 70 and 130 days of gestation [15], with the lower end of this range corresponding to early brain development and the upper end marking the latest stage at which severe structural defects are still likely to occur. For the earliest case with hydranencephaly observed in August 2024 identified in this study, which would have been conceived at the beginning of the year, it is more plausible that infection occurred closer to the upper limit (early April) of this vulnerability window rather than at its onset. This assumption is supported by the fact that winter meteorological conditions in Northern Europe are generally unfavourable to *Culicoides* activity and to viral replication within the vector, making early-season transmission events less likely. Therefore, the first vertical transmission resulting in foetal infection and subsequent hydranencephaly would most probably have occurred as soon as vector activity and viral replication became feasible, even if at relatively low levels, rather than immediately at the beginning of gestation. Considering the meteorological conditions from February to April, as well as the environmental requirements for the vectors [8,9] and for viral replication within the vector [7], it is plausible that the virus was circulating during the winter in certain farms, either indoors or outdoors. Monitoring the disease through abortion cases can thus provide additional insight into the epidemiology of bluetongue under conditions of Northern Europe. Consequently, the virus did not begin circulating in July 2024 at week 28, as the first PCR-positive case [1] might suggest; rather, it was already present several months earlier in the year since February–April 2024 or even earlier without this being detected and at a low level. This would explain the sudden occurrence of abortions infected with BTV-3 throughout the territory without observing any spatial evolution. It will be useful to support this finding by a retrospective serological monitoring of domestic ruminants.

Climate variation and longer-term change may alter *Culicoides* phenology and the seasonal window for BTV transmission in Europe; evidence is mixed across species and regions, so we note this as a consideration for future risk assessment. Several hypotheses can be proposed to explain the presence of the virus in herds where some foetal infections were undetected during the first months of the year. One possibility is that infection occurred when traders loaded the animals or due to vehicles containing infected *Culicoides*. However, no bovine purchases from the northern part of the country were reported in these herds in 2024, where the virus had previously been detected in 2023, making this scenario unlikely. Another possibility is that infected *Culicoides* could have arrived through natural dispersal before the onset of winter [24], with subsequent low-level viral circulation maintained under the prevailing winter conditions. The latter scenario is considered the most plausible explanation for the early presence of the virus in these herds.

Monitoring the outbreak through the reporting of abortion cases represents an effective surveillance approach, as it provides data daily, in contrast to occasional or winter surveillance schemes that only allow a retrospective assessment of the epidemic. However, the decision by the veterinary authorities to discontinue funding for BTV PCR testing in cases of clinical suspicion from October onwards limited data collection during the later stages of the epidemic. Fortunately, many veterinarians and farmers chose to maintain diagnostic testing at their own expense, enabling the continuation of surveillance, albeit in a more limited form.

Differences in malformation rates between beef and dairy systems may reflect variations in management, exposure, or genetic susceptibility. The observed variation across gestational stages also merits further investigation.

Preventive vaccination remains the most effective method to mitigate reproductive losses due to BTV. However, in Belgium, the lack of an anticipatory campaign and the unavailability of a BTV-3 vaccine until May 2024 allowed the virus to establish and cause important economic losses. Lessons from BTV-8 control in 2008 show that early mandatory and comprehensive vaccination is critical [27]. Considering the demonstrated pathogenicity of BTV-3, vaccination strategies should no longer rely solely on passive surveillance triggers. Moreover, the recurrent emergence of new serotypes and the risk of transplacental transmission leading to congenital malformations further underscore the importance of strategic vaccination planning. Current vaccine platforms, often serotype-specific, are not always available at the onset of outbreaks. There is an urgent need to develop safer, effective, and multivalent vaccines that can provide protection against circulating and emerging serotypes. Strategic vaccination remains a crucial tool to prevent and control bluetongue outbreaks, particularly in endemic and high-risk regions [16,28]. To reduce the vulnerability window at the start of future outbreaks, preparedness should include investment in next-generation vaccine technologies and proactive deployment strategies tailored to high-risk areas.

## 5. Conclusions

This study provides the first field-based evidence in Belgium that bluetongue virus serotype 3 (BTV-3) can cross the placental barrier in cattle inducing abortions and congenital malformations of the central nervous system as previously described for other orbiviruses as BTV-8 [17,29]. Notably, our findings indicate that BTV-3 was already circulating silently several months prior to its official detection in adult ruminants. These results highlight the critical added value of integrating abortion monitoring into early warning systems for arboviral emergence.

Given the recurrent emergence of novel BTV serotypes and the demonstrated teratogenicity of BTV-3, preparedness strategies must evolve. The continued emergence of new BTV serotypes underscores the need for strategic investment in broad-spectrum vaccine development, refined vector ecology modelling, and climate-informed forecasting tools. Such a multidisciplinary and pre-emptive approach will be essential to mitigate the reproductive, economic, and animal health consequences of bluetongue virus in an era of accelerating climatic change.

## Figures and Tables

**Table 1:** Distribution of the number of herds and abortion cases monitored.

	Number of Herds with Minimum One Reported Abortion Cases	Nb of Reported Abortion Cases	Average Number of Aborted Foetuses by Herds
2021	2341	4646	1.98
2022	2136	3997	1.87
2023	2239	4301	1.92
2024	2643	5751	2.18
TOTAL	9359	18,695	2.00

**Table 2:** List of pathogens included, and diagnostic methods applied in the standardised panel of analyses.

Pathogens	Foetus		Foetal Serum	Maternal Serum
	Samples	Methods	Methods	Methods
*Anaplasma phagocytophilum* *Brucella* spp.	Spleen Abomasal fluid	PCR *** Culture		SAW/ELISA Ab
*Campylobacter foetus* spp.	Abomasal fluid	Culture		
*Coxiella burnetii*	Abomasal fluid	PCR **		ELISA Ab
*Listeria monocytogenes*	Abomasal fluid	Culture		
Mycotic agents	Abomasal fluid	Culture		
Opportunistic bacteria	Abomasal fluid	Culture ^$^		
*Salmonella* spp.	Abomasal fluid	Culture		
BTV (Serotype 3 or 8)	Spleen	PCR *		
*Neospora caninum*	Brain	PCR	ELISA Ab	ELISA Ab
*Schmallenberg* virus	Brain	PCR *		
BoHV-4	Spleen	PCR		ELISA Ab
BVDV	Spleen	ELISA Ag		ELISA Ab
*Leptospira* serovar Hardjo	Spleen	PCR *		ELISA Ab

Legend: PCR, polymerase chain reaction; Ab, antibody; Ag, antigen; SAW, sero-agglutination of Wright; BVDV, bovine viral diarrhoea virus; BTV-8, bluetongue virus serotype 8; BoHV-4, bovine herpesvirus 4; ^$^, only the presence of a pure culture on blood agar is indicative of opportunistic bacteria; *, applied only if there is a suspected case (i.e., congenital abnormalities); **, applied only on foetuses older than 6 months; ***, applied only from April to December during the vectorial activity of the ticks.

**Table 3:** Monthly distribution of BTV PCR results from aborted foetal spleens stratified by the presence of CNS congenital lesions.

	Absence of Congenital Lesions of the CNS				Presence of Congenital Lesions of the CNS			
Month	Without PCR	With Negative PCR	With Positive PCR	Total	Without PCR	With Negative PCR	With Positive PCR	Total
January 2024	566	3		569		8		8
February 2024	521	4		525		11		11
March 2024	405	10		415		11		11
April 2024	350	9		359		18		18
May 2024	238	4		242		7		7
June 2024	180	1		181		5		5
July 2024	192	4		196		5		5
August 2024	545	27	42	614		4	12	16
September 2024	585	17	44	646	111	13	96	220
October 2024	361	19	50	430	137	6	46	189
November 2024	262	15	20	297	134	4	12	150
December 2024	334	36	4	374	230	11	22	263
Total	4539	149	160	4848	612	103	188	903

**Table 4:** Distribution of the BTV PCR results according to the congenital lesions of the CNS and in function of the gestational stage of cattle (i.e., the estimated age of the foetus at the moment of the abortion).

	Absence of Congenital Lesions				Presence of Congenital Lesions			
Estimated Gestational Stage (Month)	Without PCR	With Negative PCR	With Positive PCR	Total	Without PCR	With Negative PCR	With Positive PCR	Total
Unknown	8	0	0	8	5	0	0	5
2	2	0	0	2	0	0	0	0
3	25	2	4	31	0	0	1	1
4	53	2	1	56	18	1	5	24
5	84	7	9	100	52	1	29	82
6	193	14	6	213	121	8	40	169
7	191	11	8	210	107	7	36	150
8	306	12	29	347	95	4	21	120
9	634	32	61	727	200	13	42	255
Total	1496	80	118	1694	598	34	174	806

**Table 5:** Distribution of the number of abortions according to the congenital lesions of the CNS and in function of the cattle breed.

	Before 01/08/2024			After 01/08/2024		
Cattle Breed	Absence of Congenital Lesions	Congenital Lesions	Total	Absence of Congenital Lesions	Congenital Lesions	Total
Unknown	512	5	517	66	25	91
Meat	10,678	197	10,875	1509	475	1984
Milk	3152	51	3203	615	264	879
Mixed	880	21	901	155	73	228
Total	15,222	274	15,496	2345	837	3182

**Figure A1:**
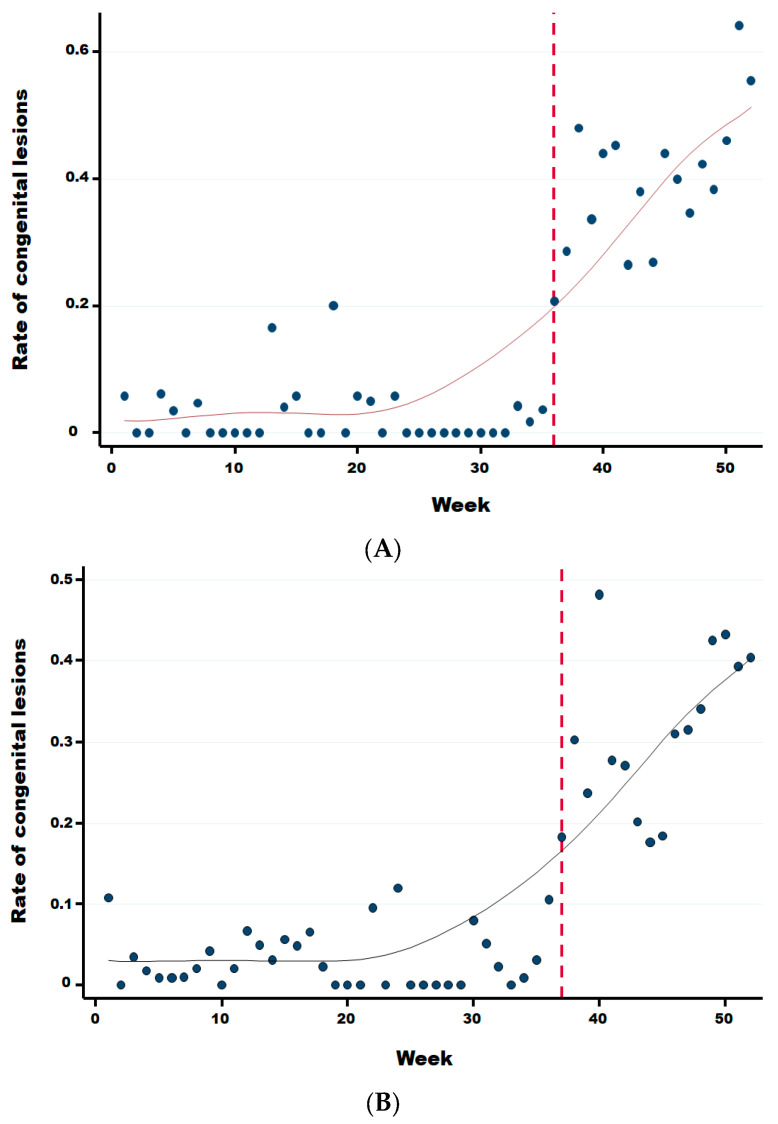

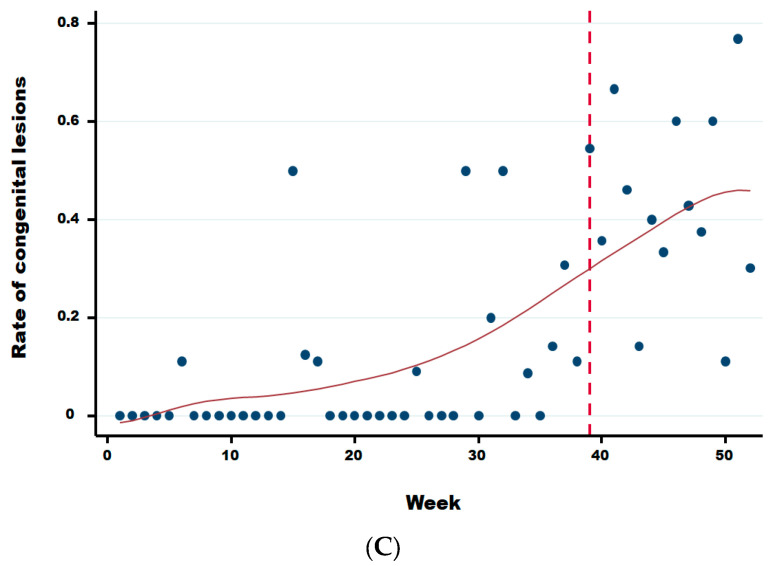
Rate of congenital lesions for dairy (**A**), meat (**B**), and mixed (**C**) cattle herds. Legend: The vertical dotted line corresponds to the starting break of the rate of congenital lesions for dairy (week 36), meat (week 37), and mixed (week 39) cattle, respectively.

## Data Availability

The data presented in this study are available on request from the corresponding author due to privacy.

## Appendix A

**Table A1:** Number of abortions with and without congenital lesions in function of the age of the foetus expressed in months.

	Abortions Without Congenital Lesions in Function of the Age of the Fœtus in Months											Abortions With Congenital Lesions in Function of the Age of the Fœtus in Months										
Month of the Year	1	2	3	4	5	6	7	8	9	?	Total	1	2	3	4	5	6	7	8	9	?	Total
1			1	11	33	71	117	124	212		569							1	1	6		8
2			5	7	23	63	76	98	249	4	525							1		10		11
3		2	1	6	13	42	56	80	212	3	415								1	10		11
4			3	9	16	45	47	81	156	2	359							2		16		18
5		2	2	7	14	24	38	43	109	3	242								2	5		7
6			2	4	15	19	29	33	78	1	181							1	1	3		5
7		1		2	13	28	42	34	75	1	196								2	3		5
8		1	4	23	76	80	65	154	209	2	614					2	8	2		4		16
9		1	7	22	43	85	76	131	279	2	646				14	50	58	42	20	35	1	220
10			13	17	17	37	55	85	206		430			1	6	15	50	42	31	44		189
11			6	7	21	39	27	64	130	3	297				4	9	29	35	31	41	1	150
12		1	7	10	24	66	64	80	119	3	374					8	35	33	46	138	3	263
Total	0	8	51	125	308	599	692	1007	2034	24	4848	0	0	1	24	84	180	159	135	315	5	903
